# Music-induced analgesia for adults and older adults during femoral arterial sheath removal after cardiac catheterization: a randomized clinical trial protocol

**DOI:** 10.1186/s12906-022-03725-8

**Published:** 2022-09-19

**Authors:** Kauanny Vitoria Gurgel dos Santos, Karena Cristina da Silva Leal, Louise Constancia de Melo Alves Silva, Kleyton Santos de Medeiros, Alexsandra Rodrigues Feijão, Maria do Carmo de Oliveira, Daniele Vieira Dantas, Rodrigo Assis Neves Dantas

**Affiliations:** 1 Graduate Program in Nursing, Department of Nursing, Federal University of Rio Grande Do Norte, Natal, Rio Grande do Norte, Brazil; 2 Department of Nursing, Federal University of Rio Grande Do Norte, Natal, Rio Grande do Norte, Brazil; 3 Graduate Program in Health Sciences, Federal University of Rio Grande Do Norte, Natal, Rio Grande do Norte, Brazil; 4 Institute of Education, Research and Innovation, Liga Contra O Câncer, Natal, RN Brazil; 5 Graduate Program in Nursing, Department of Nursing, Federal University of Sergipe, São Cristóvão, Sergipe, Brazil

**Keywords:** Cardiac catheterization, Nursing care, Pain management, Music therapy, Endovascular procedures

## Abstract

**Background:**

Cardiovascular diseases cause the death of 17.5 million people every year. Cardiac catheterization is an invasive diagnostic exam that allows treatment followed by the examination and can cause some complications such as pain. From this perspective, music has alleviated suffering and promoted pain relief for patients. This study aims to evaluate the effectiveness of music therapy to relieve pain in adults and older adults during femoral arterial sheath removal after cardiac catheterization.

**Methods:**

This is a randomized controlled clinical trial, with two arms and a single-blind design to be carried out with 68 patients equally allocated into control and experimental groups. The intervention will be applied with the use of headphones without any musical transmission in the control group or with the patient’s musical preference in the experimental group with sound intensity of 60 dB. These patients will be evaluated in three moments: immediately before, during and 15 min after the painful procedure. The primary outcome includes reduction of pain intensity verified by the Visual Analogue Scale and the secondary outcome corresponds to improvement of vital signs and vocal and facial pain expressions.

**Discussion:**

This study will allow by testing a non-pharmacological strategy to relieve pain during femoral sheath removal after cardiac catheterization, having its parameters evaluated at three moments: immediately before (30 min), during the procedure and 15 min after the painful procedure. It also enables the use of a low-cost, potentially effective resource that makes nursing care more humanized by improving user satisfaction with the service provided, in addition to reducing the need for post-procedure analgesics.

**Trial registration:**

This study is registered on the Brazilian Clinical Trials Registry (REBEC) platform under number RBR-3t3qwp7 (05/04/2022) and was approved by the Research Ethics Committee of the Federal University of Rio Grande do Norte under CAAE 52,586,521.8.0000.5537 (11/11/2021).

## Background

Cardiovascular diseases (CVD) are the leading cause of death worldwide, causing the death of 17.5 million people every year according to the World Health Organization (WHO) [1]. As one of the greatest public health problems, acute coronary syndrome stands out through the occurrence of Acute Myocardial Infarction—with or without ST segment elevation—and stable angina, which affects about 85.6 million adults with at least one type of the disease, only in the United States [2].

Acute coronary diseases are diagnosed through electrocardiogram examination, analysis of clinical signs and cardiac enzymes and are confirmed by hemodynamic evaluation, cardiac catheterization. Also known as coronary angiography or cine-angiography, cardiac catheterization is an invasive diagnostic test performed to analyze coronary vessels or heart muscles through the insertion of catheters by arterial puncture in the arms or legs to reach heart chambers. Thus, this therapeutic approach provides accuracy in anatomical and functional visualization, which allows the treatment to be carried out followed by the examination [3].

It was observed that the establishment of more elaborate processes in hemodynamics laboratories enables the appearance of complications in patients undergoing endovascular procedures. Among them, those that stand out most frequently are pseudoaneurysms, hematomas, distal embolization, bleeding at the insertion site, and arterial thrombolysis [4]. However, acute pain is the most recurrent complaint of patients undergoing endovascular procedures, either in the puncture site, in the back, thoracic and lumbar region, or even headache. The painful phenomenon may be associated with limb immobilization, bed restriction, or due to puncture-related vascular trauma or complications during the procedure [5].

Pain is related to both a sensory and an emotional experience [6]. In this sense, many tools have been used in the scientific and clinical environment to measure pain, considering that it is considered the fifth vital sign by the American Pain Society and its evaluation should be mandatory [7].

The measurement of this phenomenon can be performed through the use of scales to assess pain in adults, namely: non-verbal pain scale (NVPS), critical care pain observation tool (CPOT), behavioral pain scale (BPS), behavioral pain assessment tool (BPAT), pain assessment tool (PAT) and COVERS scale, behavioral pain indicators scale (ESCID) [8]. The Visual Analogue Scale (VAS) is understood as the simplest and most universal scale that enables the application of a self-assessment method for the patient from the absence of pain to unbearable pain, ranging from zero to ten, from one to three being considered mild pain, four to seven moderate and eight to ten severe pain [9].

It is up to the nurse to assist in pain management throughout the nursing process, using to assess pain, pharmacological and non-pharmacological therapies and the control of patient responses. Thus, the implementation of non-pharmacological strategies is considered as complementary, helping to relax, improve sleep patterns and relieve pain [10]. Thus, nursing has used Integrative and Complementary Practices in Health (ICPS) as a new method of patient care for pain relief, namely: acupuncture, aromatherapy, cryotherapy, phytotherapy, hydrotherapy, homeopathy, massage, meditation, music therapy, reiki and therapeutic touch [11].

The use of music therapy also collaborates in the therapeutic treatment with physiological effects related to changes in metabolism, regulation of respiratory rate, release of adrenaline, variations in Blood Pressure (BP), reduction of fatigue and increase in sensory stimuli. Therefore, it is an accessible therapeutic tool, easy to use, with no side effects, allowing wide use in different therapeutic scenarios [12]. From this perspective, interventions with music alleviate suffering and pain in patients by promoting positive auditory stimuli that camouflage adverse sounds, improve emotional health and induce a feeling of hope and well-being during treatment [13].

Similarly, a retrospective study carried out with 99 patients in China sought to evaluate the effects of music on anxiety, pain and depression of patients after coronary artery bypass graft surgery. The research describes significant reduction in the numerical pain scale (*p* < 0.05) after 30 min of musical intervention, when compared with results using the same scale before intervention and when comparing the experimental group with the other study groups: rest without music and standard service care [14].

In addition to these important variables, a quasi-randomized Controlled Clinical Trial described the effects of natural sounds on anxiety and physiological parameters such as systolic, diastolic and mean blood pressure, Heart Rate (HR) and oxygen saturation in 130 subjects undergoing coronary angiography. Positive and statistically significant results (*p* < 0.05) have been reported in reducing anxiety and the physiological parameters associated with it, and it is therefore considered an effective intervention, with no side effects [15].

Given the above, the questions are: is music effective in relieving pain during arterial sheath removal after cardiac catheterization via the femoral route? What physiological changes are promoted by the use of this strategy? Does the use of this technology influence the outcome of the Visual Analogue Scale (VAS) of pain in these patients?

### Objective

To evaluate the effectiveness of music therapy to relieve pain in adults and older adults during femoral arterial sheath removal after cardiac catheterization.

## Methods/design

This study is registered on the Brazilian Clinical Trials Registry (REBEC) platform under number RBR-3t3qwp7 (05/04/2022) and was approved by the Research Ethics Committee of the Federal University of Rio Grande do Norte under CAAE 52,586,521.8.0000.5537 (11/11/2021). This protocol complies with the Standard Protocol Items for Randomized Trials (SPIRIT) [16].

### Trial design

This is a protocol of a Randomized Controlled Clinical Trial (RCT) with two arms and single-blind intervention design, which compares the effects of music therapy to relieve pain in adults and older adults during femoral arterial sheath removal after cardiac catheterization with standard interventions already used by the service for this same procedure.

### Population

The population will be composed of individuals who will undergo cardiac catheterization at the largest hemodynamics service of the state of Rio Grande do Norte, located in Natal, state of Rio Grande do Norte, Brazil, with the need to remove the arterial sheath via the femoral route. The sample will be random and simple probability.

### Eligibility and recruitment criteria

**Fig. 1:**
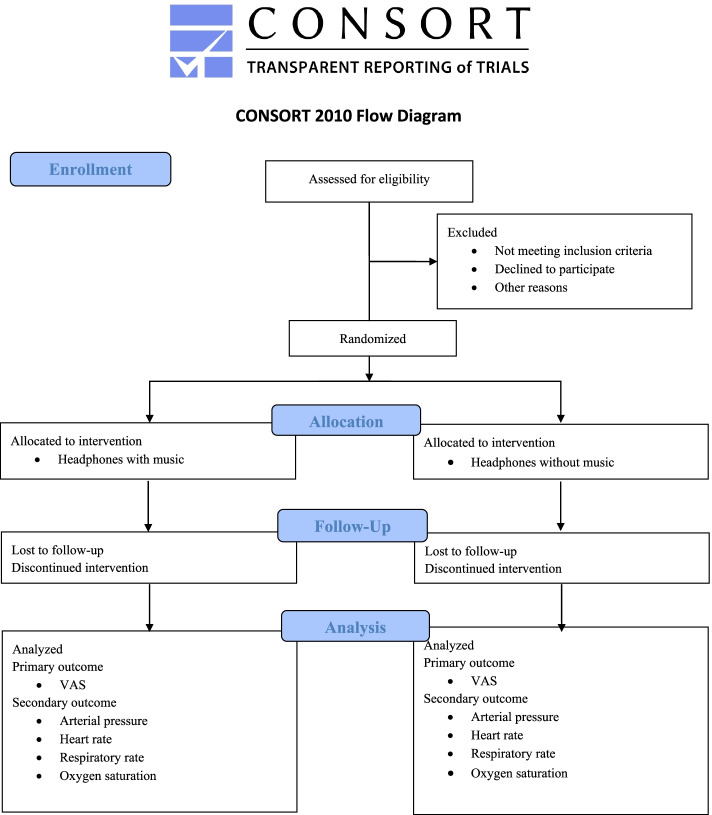
Flow diagram of survey participants adapted from CONSORT (2010) [17]

The following inclusion criteria will be adopted: patients aged 18 years or older admitted to hemodynamics for elective cardiac catheterization, with the need to remove the arterial sheath via the femoral route. Those who do not like music, who have previously undergone the procedure, those with chronic pain, diagnosis of arboviruses, fibromyalgia, previous analgesia, individuals with neurological and/or cognitive problem that compromise the understanding and use of the scale and individuals with hearing impairment will be excluded. Figure 1 shows the study flow.

#### Interventions

The Control Group (CG) will be composed of 34 patients allocated in a simple random way with clinical indication of performing cardiac catheterization. These patients will receive the standard care already adopted by the service, in addition to the application of headphones without any musical transmission for the removal of the arterial sheath via the femoral route in a timely manner and will remain with the headphones for 15 min after the end of the painful procedure.

The Experimental Group (EG), in turn, will be composed of 34 patients allocated in this group in a simple random way, who will receive the same care as the previously mentioned group, in addition to the application of headphones which will transmit the patient's musical preference during the performance of the painful procedure up to 15 min after its completion. The headphones will be used in a complementary way and not as a substitute for the care already adopted by the service.

#### Questionnaire

At first, the principal researcher will apply a form previously prepared with participants of both groups containing the following sociodemographic data: date of birth, age, sex, color, marital status, schooling, profession, family income and musical preference.

At this time, the initial part of the second previously prepared form will also be applied, containing the following clinical data: comorbidities, reason for performing cardiac catheterization and medications in use. After completing this part, blood pressure, heart and respiratory rate vital signs will be measured, as well as information regarding oxygen saturation, presence or absence of bodily signs and symptoms of pain such as sweating, pallor, vocal or face expression or pain. These data will be recorded in the second previously prepared form and repeated during the intervention and 15 min after the end of the painful procedure.

This second form also contains information regarding the French number of the arterial sheath, the professional responsible for removing it, if there were complications during the painful procedure and what these complications were; if there were complications after the procedure and what these complications were; how long the arterial compression lasted after the intervention; if there was need for analgesia after the procedure and the time to receive the hemodynamic discharge. Finally, the Visual Analog Pain Scale will be applied to identify pain intensity.

## Results

### Primary outcome

The primary outcome expected is reduction in pain intensity through the Visual Analogue Scale (VAS) [17] and reduction in the following physiological parameters: blood pressure, heart and respiratory rate, in addition to the improvement of oxygen saturation levels through the application of the patient's musical preference using headphones during the removal of the arterial sheath via the femoral route after cardiac catheterization.

### Secondary outcome

The secondary outcome expected is absence of bodily signs and symptoms of pain verified through the absence of the following parameters: sweating, pallor and facial expression of pain, in addition to reduction of the intensity levels of vocal expressions of pain, namely: screaming, crying, groaning, verbal report of pain or their absence through the application of the patient's musical preference using headphones during the removal of the arterial sheath via the femoral route after cardiac catheterization.

### Screening

Immediately before carrying out the procedure, the first researcher will recruit the possible subjects and explain the main study objective, the possible risks and benefits involved in the study and emphasize that he/she may withdraw from participating in the research at any stage of it.

If there is affirmative answer, this first researcher will apply the inclusion and exclusion criteria to select the individuals who will compose the sample. Then, they will be asked to read and sign the Free and Informed Consent Form (FICF) to guarantee their participation in the study.

### Follow-up

Table 1 describes the adapted SPIRIT schedule with appropriate enrollment times, interventions and participant assessments.

**Table 1:** Standard protocol items: adapted recommendations for interventional trials (SPIRIT) schedule: enrollment, interventions and evaluations

STUDY PERIOD						
	**Enrolment**	**Baseline**	**Post-allocation: Allocation, Interventions, Follow-up**			
**TIMEPOINT**			**Allocation**	**P1**	**P2**	**Follow-up (F1)**
**ENROLMENT:**						
Eligibility criteria	X					
Recruitment	X					
Initial assessment	X					
Informed consent	X					
Allocation			X			
**INTERVENTIONS:**						
* Headphones* with no musical transmission (Control group)					X	X
* Headphones* with the patient’s musical preference (Experimental group)					X	X
**ASSESSMENTS:**						
Sociodemographic data		X		X		
Clinical data		X		X	X	X
VAS						X

Legend:

P1: moment immediately before arterial sheath removal (30 min before)

P2: moment during arterial sheath removal

F1: moment after arterial sheath removal (15 min later)

*VAS *Visual Analog Scale

Patients participating in the study will be monitored throughout their stay in the hemodynamic admission/recovery room. In this sense, after carrying out the cardiac catheterization and returning to the aforementioned room, the patient will be approached in an attempt to recruit and select the sample. After applying the inclusion and exclusion criteria, patients will be asked to sign the informed consent form, guaranteeing their participation in the study and allocation to control or experimental groups. At this initial moment, sociodemographic data and the first part of clinical data will already be completed, initiating their follow-up.

After the initial 30 min, if the patient's blood pressure is within acceptable limits, the health professional will remove the arterial sheath via the femoral route. At this point, the second part of the instrument referring to clinical data will be completed, continuing the follow-up.

Finally, after arterial sheath removal and after 15 min of arterial compression according to the service protocol, the headphones will be removed and the third and last part of the instrument referring to clinical data will be completed and finished with the VAS application for the identification the pain intensity. After performing the compressive dressing, the individual will spend four hours keeping the limb at rest until discharged from the unit, at which time follow-up will be completed.

### Sample size

The sample size calculation was performed using the G Power software, version 3.1.9.2, with Cohen effect of 0.50, test power of 0.80 and significance level of 5%, establishing N of 34 patients per group, experimental and control, totaling 68 patients.

### Allocation randomization and hiding

Patients will be randomly allocated into two groups: Control Group (CG) and Experimental Group (EG). Their allocation will be guaranteed through the “Research Randomizer” website so that all patients have an equal chance of becoming part of one group or another.

#### Blinding

The study will have the participation and involvement of two researchers. Researcher 1 (R1) will be responsible for the recruitment and initial selection of individuals that will compose the sample, as well as for guaranteeing their participation by signing the Free and Informed Consent Form (FICF), performing the randomization and allocation of patients and application of headphones in both groups. The second researcher will be responsible for applying the data collection instruments during the intervention and at the end of the painful procedure. Thus, the second researcher, service health professionals and patients will not know to which groups patients will be allocated.

#### Data management

First, two researchers (R1 and R2) will be trained to perform data collection, in accordance with the study protocol. The information will be collected in the patient's admission/recovery room and stored in database previously built in Microsoft Excel. Subsequently, data will be exported to the Statistical Package for Social Science Version 18.0 software (SPSS Version 20.0) in order to perform descriptive and inferential analyses. Therefore, data will be presented through tables, charts and figures.

It is worth mentioning that losses and dropouts will be carefully recorded in order to ensure study reliability. Therefore, whoever withdraws from participating in the research will be replaced by the next participant who meets the inclusion and exclusion criteria, being included in the group referring to the withdrawal until reaching the previously calculated sample number. All information will be stored in a safe place by the responsible researcher for a period of five years and will only be presented in scientific publications, preserving the anonymity of participants.

#### Data extraction and statistical analysis

Data will be described in a database previously built in Microsoft Excel and exported to the Statistical Package for Social Science Version 18.0 software (SPSS Version 20.0), where descriptive and inferential analyses will be performed. The Kolmogorov–Smirnov test will be used to assess the normality of variables. For the evaluation of variables, the Chi-square (χ^2^) or Fisher's Test will also be used. Other tests that may be necessary will also be adopted. Results will be presented through tables, charts and figures, adopting significance level of 5% (*p* = 0.05).

## Discussion

Coronary Artery Disease (CAD) has coronary angiography as the gold standard test for its diagnosis [18], in addition to Percutaneous Coronary Intervention as one of the main forms of treatment [19] using, above all, the femoral access route. However, some complications may be associated with the use of this route, such as bleeding, bruising, arterial occlusion, prolonged hospital stay or even discomfort due to the strategy used for hemostasis, rest time, and pain in the lumbar, back, or in the puncture site [18, 19].

Therefore, randomized clinical trials have used VAS to measure acute and chronic pain intensity after cardiac catheterization. This scale is subjective and graded from zero to ten, with zero representing no pain and ten corresponding to unbearable pain [18, 20].

Music is a strategy that has been used by studies with the purpose of acting in the alleviation of pain and anxiety and improvement of physiological parameters in percutaneous coronary procedures. An RCT was performed in Turkey with 62 patients who would undergo coronary angiography via the femoral route. The music was heard through a CD prepared by a specialist at volume 20 for 45 min before the procedure. This intervention was described as effective, especially in reducing anxiety, diastolic blood pressure, respiratory rate and pain after the procedure. In addition, the need for analgesics and sedation was also reduced in the experimental group [21].

However, musical intervention can be used in different ways and be equally effective. Another study conducted at the University of Aksaray Training and Research Hospital, with 171 subjects, used instrumental music with the flute through headphones for about 60 to 80 beats per minute to induce relaxation. This type of music was selected by a specialist in the field and, at the end of the intervention, significant reduction in systolic blood pressure (*p* < 0.05) and pain levels (*p* < 0.001) were described in the experimental group [22].

The literature described through an RCT meta-analysis and in quasi-experimental studies, the use of music in percutaneous coronary procedures when assessing pain, anxiety, discomfort, HR and BP of participants. It was observed that musical intervention is effective in reducing anxiety, but it does not apply for the assessment of pain, HR and BP, whose results indicate that musical intervention was not superior to routine care. The meta-analysis concluded that studies included in the sample are heterogeneous and suggested performing new high-quality RCTs with music intervention to confirm these findings [23].

In this context, this RCT intends to evaluate the effectiveness of music therapy to relieve pain in adults and older adults during femoral arterial sheath removal after cardiac catheterization.

This study advances knowledge by testing a non-pharmacological strategy to relieve pain during femoral sheath removal after cardiac catheterization, having its parameters evaluated at three moments: immediately before (30 min), during the procedure and 15 min after the painful procedure. It also enables the use of a low-cost, potentially effective resource that makes nursing care more humanized by improving user satisfaction with the service provided, in addition to reducing the need for post-procedure analgesics. However, it is limited by being developed in only one university hospital located in Brazil, therefore understanding the reality of this location.

### Ethics and disclosure

This clinical trial was approved by the Research Ethics Committee of the Federal University of Rio Grande do Norte under CAAE 52,586,521.8.0000.5537 (11/11/2021) and CEP 5.100.469 (11/11/2021), in addition to being registered on the Brazilian Registry of Clinical Trials (REBEC) platform under number RBR-3t3qwp7 (05/04/2022).

The participation of individuals will be on a voluntary and non-profit basis, being guaranteed through the signature of the FICF. Data provided are confidential and will only be disclosed at conferences or scientific publications, anonymously, without disclosing any information that allows patient identification. Results will be kept by the responsible researcher in a safe place and for a period of 5 years.

## Data Availability

Data sharing is not applicable to this article as no datasets were generated or analysed during the current study.

## Abbreviations

BPAT: Behavioral Pain Assessment Tool
ESCID: Behavioral Pain Indicators Scale
BPS: Behavioral Pain Scale
BP: Blood Pressure
REBEC: Brazilian Clinical Trials Registry
CVD: Cardiovascular Diseases
ICPS: Complementary Practices in Health
CG: Control Group
CAD: Coronary Artery Disease
CPOT: Critical Care Pain Observation Tool
EG: Experimental Group
FICF: Free and Informed Consent Form
HR: Heart Rate
NVPS: Non-verbal Pain Scale
PAT: Pain Assessment Tool
RCT: Randomized Controlled Clinical Trial
R1: Researcher 1
R2: Researcher 2
SPIRIT: Standard Protocol Items for Randomized Trials
SPSS: Statistical Package for Social Science
VAS: Visual Analogue Scale
WHO: World Health Organization
